# Identification, characterization and spatiotemporal expression analysis of the *FKBP* family genes in *Locusta migratoria*

**DOI:** 10.1038/s41598-023-30889-1

**Published:** 2023-03-10

**Authors:** Neng Zhang, Shiqian Feng, Ye Tian, Ling Zhuang, Gan Cha, Saiya Duan, Hongmei Li, Xiangqun Nong, Zehua Zhang, Xiongbing Tu, Guangjun Wang

**Affiliations:** 1 State Key Laboratory for Biology of Plant Diseases and Insect Pests, Institute of Plant Protection, Chinese Academy of Agricultural Sciences, Beijing, 100193 China; 2 Scientific Observation and Experimental Station of Pests in Xilingol Rangeland, Ministry of Agriculture and Rural Affairs, Xilinhot, 026000 China; 3 Bayannur Forestry and Grassland Development Center, Bayannur, 015000 China; 4 MARA-CABI Joint Laboratory for Bio-Safety, Institute of Plant Protection, Chinese Academy of Agricultural Science, Beijing, 100193 China

**Keywords:** Biochemistry, Computational biology and bioinformatics, Evolution, Zoology

## Abstract

FK506 binding proteins (FKBPs) are a highly-conserved group of proteins known to bind to FK506, an immunosuppressive drug. They play different physiological roles, including transcription regulation, protein folding, signal transduction and immunosuppression. A number of *FKBP* genes have been identified in eukaryotes; however, very little information about these genes has been reported in *Locusta migratoria*. Here, we identified and characterized 10 *FKBP* genes from *L. migratoria*. Phylogenetic analysis and comparison of domain architectures indicated that the *LmFKBP* family can be divided into two subfamilies and five subclasses. Developmental and tissue expression pattern analysis revealed that all *LmFKBPs* transcripts, including *LmFKBP46*, *LmFKBP12*, *LmFKBP47*, *LmFKBP79*, *LmFKBP16*, *LmFKBP24*, *LmFKBP44b*, *LmFKBP53*, were periodically expressed during different developmental stages and mainly expressed in the fat body, hemolymph, testis, and ovary. In brief, our work depicts a outline but panoramic picture of *LmFKBP* family in *L. migratoria*, and provides a solid foundation to further investigate the molecular functions of *LmFKBPs*.

## Introduction

FK506 binding proteins are members of the peptidyl-prolyl cis–trans isomerases (PPIase) family, named for their binding of the immunosuppressive drug, FK506 (Tacrolimus)^1,2^. FKBPs of different sizes and functions have been identified in the archaea and eukaryotes. In archaea, FKBPs were reported to help proteins to refold under temperature stresses^3^. In *Saccharomyces cerevisiae*, the FKBP12-FK506 complex was identified to selectively bind to calcineurin (CaN) and inhibited its activity, thereby participating in the signal transduction process^4^. In plants, FKBPs have been reported to be involved in responses to environmental stress^5^, binding transcription factors^6,7^, regulating plant growth and development as well as other physiological metabolic processes^8,9^. In animals, FKBPs have been reported to bind to DNA^10,11^, participate in the regulation of multiple key signal pathways by regulating autophagy^12^, bind to RyRs (Ryanodine receptors)^13^ and mTOR (mammalian target of rapamycin protein)^14^, and participate in different important life processes^15^. In insects, FKBPs have been reported to participate in the regulation of embryonic development^16^, regulate the development of eyes and wings^17^, and mediate resistance to virus^18^.

The migratory locust, *Locusta migratoria*, with the characteristics of large food consumption, long migration distance, rapid development and strong reproductive capacity, is one of the main pests endangering food security^19^. At the same time, locusts also have a strong immune defense response. If it can directly inhibit the immune defense of them, it will help to improve the effect of locust control. Studying the LmFKBPs could provide insight into the immune mechanism of insects. However, a systematic identification and functional analysis of FKBPs in *L. migratoria* is still absent. Fortunately, some works have been done on the identification and expression pattern analysis of chemosensory protein genes^20^ and cytochrome P450 monooxygenases (P450s) genes^21^ in locusts, which will be of great reference for the completion of our paper. In our study, we amplified 10 full-length *LmFKBPs* complementary DNA (cDNA) sequences, and analyzed their evolutionary relationships, protein characteristics, and spatiotemporal expression patterns. These results help illuminate the biological functions of *LmFKBPs* and also explore their potential as new targets for the management of *L. migratoria*.

## Materials and methods

### Insect rearing

The *L. migratoria* population used in the present study and the rearing methods were based on Li et al.^22^. Specifically, the rearing temperature is 30 ± 2 °C, with a relative humidity (RH) of 60 ± 5% and a photoperiod of 14:10 (light:dark) hours. After hatching, larvae were placed in well-ventilated rearing cages (60 cm × 50 cm × 70 cm) in the insectarium at the Institute of Plant Protection, Chinese Academy of Agricultural Sciences and raised insects on fresh wheat seedlings.

### Gene acquisition and bioinformatics analysis

The homologous DNA sequences of *LmFKBPs* were obtained based on BLAST searches (E-value < = 0.001) through BioEdit 7.0.9 against *Drosophila melanogaster DmFKBP12* gene sequence (GenBank: AAL48728.1), using both the transcriptome and the whole-genome databases of *L. migratoria* (http://www.locustmine.org/). Conserved Domain Database (CDD; https://www.ncbi.nlm.nih.gov/cdd/) and Simple Modular Architectural Research Tool (SMART; http://smart.embl-heidelberg.de/) were used to predict the conserved domains in LmFKBPs. ProtParam programed on the Expasy website (https://www.expasy.org/) was used to perform molecular weight and theoretical Isoelectric point calculation; WoLF PSORT website (https://wolfpsort.hgc.jp/) was used to predict the subcellular location. For phylogenetic analysis, multiple protein sequence alignment was performed using MUSCLE and the phylogenetic tree was constructed using MEGA 7 software with the neighbor-joining method and bootstrap analysis with 1000 replications^23^.

### RNA extraction and cDNA synthesis

Total RNA was extracted using the TRIzol reagent (ambion) following the manufacturer’s protocol. Samples were taken at different developmental stages (eggs, the first, second, third, fourth, and fifth instars and adults) and from different tissues (integument, head, leg, hemolymph, fat body, midgut, testis, and ovary of the adults). Total RNA concentrations were determined using a Nano-300 spectrophotometer. First-strand cDNA was prepared from total RNA by reverse transcription using the abm 5 × All-In-One MasterMIx Reverse Transcription Kit.

### Gene cloning and sequencing

For verification of the sequence, the cDNA was amplified, ligated into a cloning vector and sequenced. The program was as follows: 95 °C for 4 min; followed by 35 cycles of 95 °C for 10 s, 57 °C for 10 s and 72 °C for 45 s; then a final extension at 72 °C for 2 min. The PCR products were examined by 1.0% agarose gel electrophoresis and purified using a DNA gel extraction kit (Tsingke Biotechnology Co., Ltd). Then the amplified products were cloned into the pClone 007 vector (Tsingke Biotechnology Co., Ltd), and positive clones were verified by sequencing at Sangon Biotech (Shanghai) Co., Ltd. The primers used are listed in Table 1.

**Table 1:** Primers used in the present study.

Genes	Primers	Primer sequences (5′–3′)	Purposes
*LmFKBP12*	LmFKBP12-F	GTTGTGATTTGTGATAGGTATTTC	Gene cloning
	LmFKBP12-R	ATTCAATGGTTATTTCAGGAG	
*LmFKBP16*	LmFKBP16-F	GGCAGCACGCTAAAAGA	
	LmFKBP16-R	AGCACTCGCATACTTCACC	
*LmFKBP24*	LmFKBP24-F	CCCTCTATGTGGGAGAAATC	
	LmFKBP24-R	CATCGTTACAGCTCATCATG	
*LmFKBP35*	LmFKBP35-F	CCCAGCGTAATGATGAACTT	
	LmFKBP35-R	CGCTGATGTGACTACTGTGC	
*LmFKBP44b*	LmFKBP44b-F	TGCGTTGGTTACATCAAGTG	
	LmFKBP44b-R	CCAGAACTCTCATTCAGTCACA	
*LmFKBP44a*	LmFKBP44a-F	CAGACTCGACTCATGGACAC	
	LmFKBP44a-R	GTGTTTGTTTAGCACCGTCA	
*LmFKBP46*	LmFKBP46-F	CGTTGTGGAGTGGAAGAGTC	
	LmFKBP46-R	CTTACAAAGATCAGCGGAACA	
*LmFKBP47*	LmFKBP47-F	CTCAGTATCGCAAGGTTCG	
	LmFKBP47-R	CCCACTCTCCCAATGTCTT	
*LmFKBP53*	LmFKBP53-F	GCTCGTTGTGTTTGGTAACC	
	LmFKBP53-R	ATGGACATCACTGAGGAGGG	
*LmFKBP79*	LmFKBP79-F	ATGGAAAGTGGCGATCAC	
	LmFKBP79-R	TTACAGAAACTTGTAACGGTAAGC	
*LmActin*	qPCR-actin-F	TCCAGCCTTCATTCTTGGGT	RT-qPCR
	qPCR-actin-R	CCTCTCAGGTGGAGCAATGA	
*LmFKBP12*	qPCR-LmFKBP12-F	GCCAGACTGTGGTTGTTCAC	
	qPCR-LmFKBP12-R	ATCTGGAGAGCACGTAAGGC	
*LmFKBP16*	qPCR-LmFKBP16-F	CAACAAACCCGGCAGCAC	
	qPCR-LmFKBP16-R	CCATCCTCCAGTGAACCCTT	
*LmFKBP24*	qPCR-LmFKBP24-F	CGTGTTGAGTCTAGCGTTCC	
	qPCR-LmFKBP24-R	CTGGAGTCGAACTGCTTGC	
*LmFKBP35*	qPCR-LmFKBP35-F	GAGGTCACTGTGCATTACCG	
	qPCR-LmFKBP35-R	TTCCTCGTTCACCATAGGCA	
*LmFKBP44b*	qPCR-LmFKBP44b-F	GGAAGCGGAAGAATCGGAAG	
	qPCR-LmFKBP44b-R	ATCACTGTCACCGTCGTCAT	
*LmFKBP44a*	qPCR-LmFKBP44a-F	TAAAGCAAAGACGGCAGACG	
	qPCR-LmFKBP44a-R	TCGTTGACCTGCCTTCTCAT	
*LmFKBP46*	qPCR-LmFKBP46-F	AAGTCGTACGAGAAGCCCAA	
	qPCR-LmFKBP46-R	TCTCCCTGCACCAAACTCTT	
*LmFKBP47*	qPCR-LmFKBP47-F	AAGCATGGTCCCTAGATGCT	
	qPCR-LmFKBP47-R	AAGTGTCCGGCCAGTAGTAG	
*LmFKBP53*	qPCR-LmFKBP53-F	TGGTCGAGCGTTAATAGGCT	
	qPCR-LmFKBP53-R	AGCTCCAGGCTGAACATTCT	
*LmFKBP79*	qPCR-LmFKBP79-F	CTCCAGACATTCCACCGGAT	
	qPCR-LmFKBP79-R	AGAAACCACCCTCAGCATCA	

### Developmental and tissue expression analysis of *LmFKBPs*

Real-time quantitative PCR (RT-qPCR) was used to determine the expression levels of *LmFKBPs* at different developmental stages and in different tissues. The *LmActin* gene was used as an internal control^20^. RT-qPCR was conducted in a QuantStudio 5 Real-Time PCR Instruments (ThermoFisher Scientific, Shanghai, China) and TB Green^®^ Premix Ex Taq TM (Takara) with a standard protocol: initial denaturation at 95 °C for 2 min, followed by annealing at 40 cycles at 95 °C for 5 s and extension at 60 °C for 30 s. Melting curve generation was performed from 60 to 95 °C. For each sample, we used three technical replicates and three biological replicates. The relative expression level was calculated using the 2^−ΔΔCt^ method^24^. The primers used are shown in Table 1.

### Statistical analysis

All the statistical analyses were conducted using SAS 9.4. One-way analysis of variation (ANOVA) followed by LSD method multiple comparisons was used to compare the relative expressions of *LmFKBPs* in different developmental stages or tissues. *P*-value < 0.05 was considered to be statistically significant.

## Results

### Cloning and characterization of FKBPs in *L. migratoria*

After blastn searches, we got similar results from transcriptome and genome da-tasets and identified 10 genes containing the FKBP-type peptidyl-prolyl cis–trans isomerase (FKBP_C) domain, forming the *FKBP* family of *L. migratoria*. The usual naming convention, adopted here, involved the addition of a prefix indicating the species to the term FKBP and a suffix reflecting the calculated molecular mass of the mature protein^25^. And these genes were named *LmFKBP12*, *LmFKBP16*, *LmFKBP24*, *LmFKBP35*, *LmFKBP44b*, *LmFKBP44a*, *LmFKBP46*, *LmFKBP47*, *LmFKBP53*, *LmFKBP79*. The lengths of these open reading frames (ORFs) ranged from 330 to 2106 bp, and *LmFKBP12* is the shortest gene with 330 bp, and *LmFKBP79* is the largest gene with 2106 bp, respectively. The molecular weights, theoretical isoelectric points, and accession numbers of all *LmFKBPs* are listed in Table 2. Furthermore, predictions of their subcellular locations showed that LmFKBP16 and LmFKBP24 are secretory proteins; LmFKBP35 and LmFKBP44b are located in the nucleus; LmFKBP12, LmFKBP44a, LmFKBP46, LmFKBP47 and LmFKBP79 are located in the cytosol; and LmFKBP53 is located in both the cytosol and nucleus (Table 2).

**Table 2:** Characterization of FKBPs in *L. migratoria.*

Genes	Blast E-value	Length (bp)	Molecular weight	Isoelectric point	Subcellular location	GenBank accession number
*LmFKBP12*	1.7E^−25^	330	11,822.47	6.71	Cytosol	OL311481
*LmFKBP16*	4.7E^−41^	441	16,075.61	9.19	Secreted	OL311482
*LmFKBP24*	7.5E^−39^	654	24,113.06	4.67	Secreted	OL311483
*LmFKBP35*	5.9E^−41^	987	35,470.28	5.50	Nucleus	OL311484
*LmFKBP44b*	1.5E^−30^	1203	43,792.59	4.97	Nucleus	OL311485
*LmFKBP44a*	2.5E^−25^	1227	43,828.96	5.32	Cytosol	OL311486
*LmFKBP46*	5.8E^−22^	1245	46,361.03	8.24	Cytosol	OL311487
*LmFKBP47*	5.8E^−52^	1275	47,426.94	5.82	Cytosol	OL311488
*LmFKBP53*	3.1E^−22^	1407	52,864.55	5.21	Cytosol and Nucleus	OL311489
*LmFKBP79*	3.2E^−19^	2106	78,513.44	4.91	Cytosol	OL311490

### Phylogenetic and classification analysis of FKBPs in *L. migratoria*

**Figure 1:**
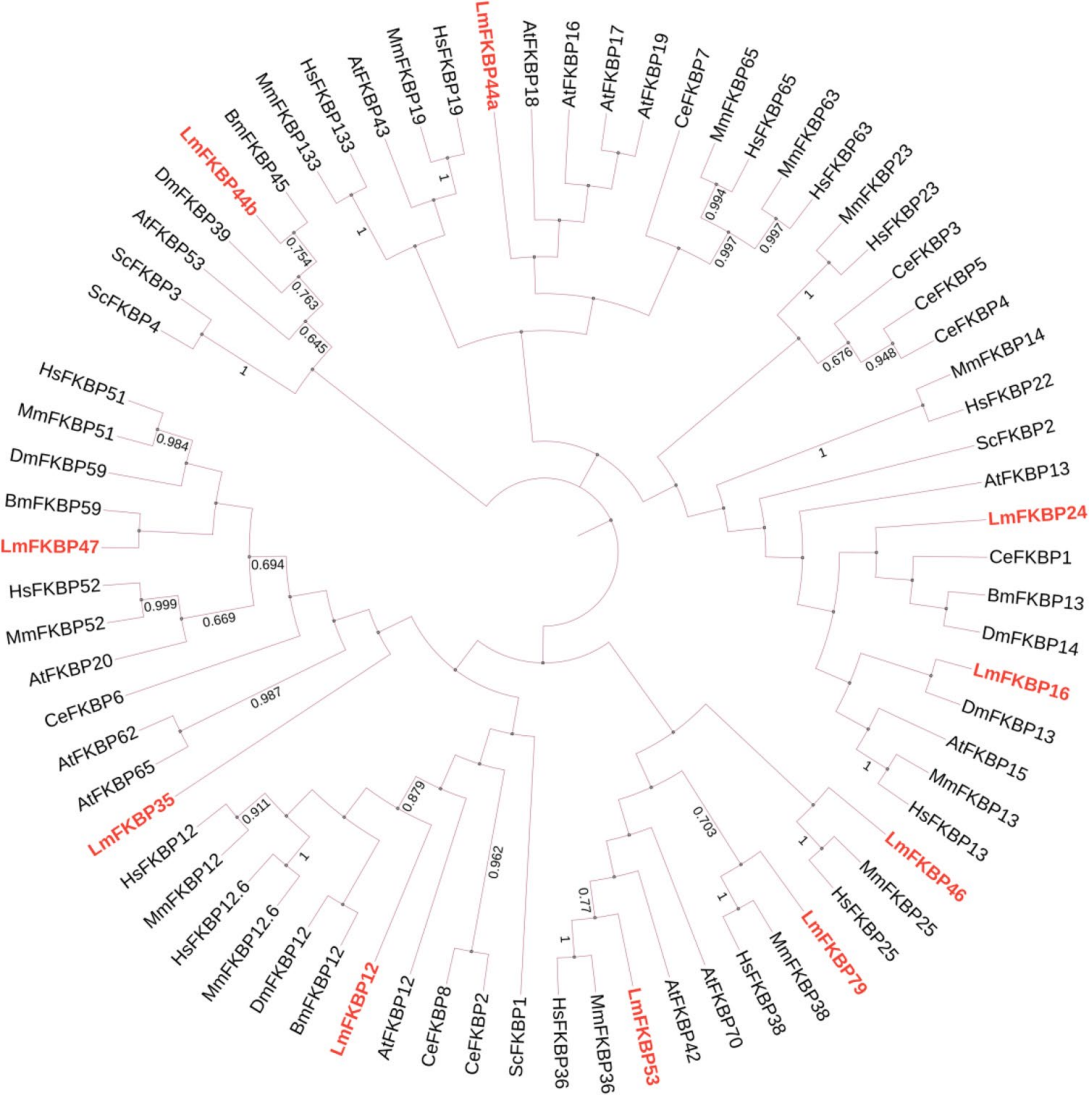
Phylogenetic analysis of FKBPs from *Saccharomyces cerevisiae* (Se), *Homo sapiens* (Hs), *Arabidopsis thaliana* (At), *Mus musculus* (Mm), *Drosophila melanogaster* (Dm), *Bombyx mori* (Bm), *Caenorhabditis elegans* (Ce), and *Locusta migratoria* (*Lm*). The UniProt accession numbers of these FKBPs are listed in Table S1.

**Figure 2:**
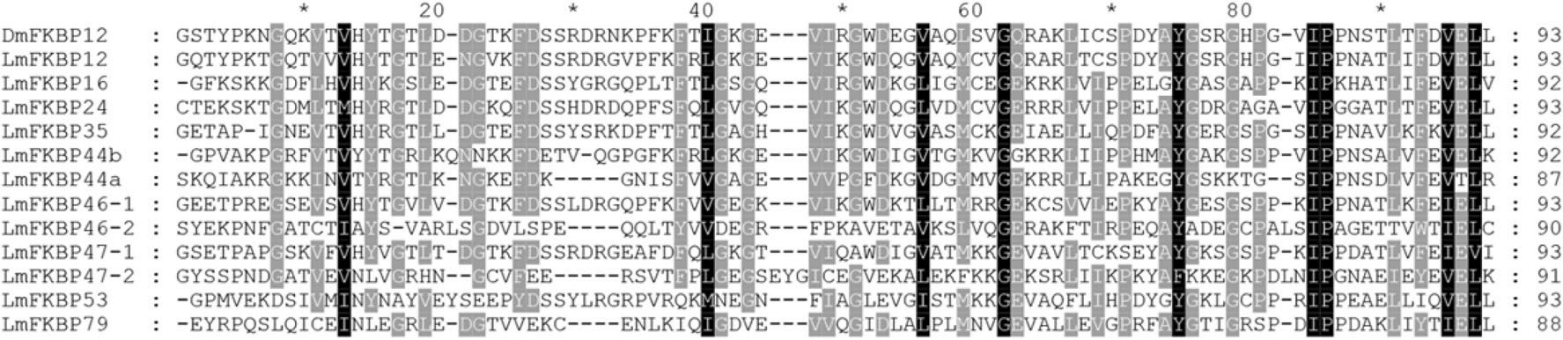
Multiple sequences alignment of FKBP_C domains. *Drosophila melanogaster* (Dm); *Locusta migratoria* (Lm). The ClustaIW program in MEGA7 software was used for multiple sequence alignment, and the GeneDoc software was used for mapping. The shaded part indicates conserved amino acids.

**Figure 3:**
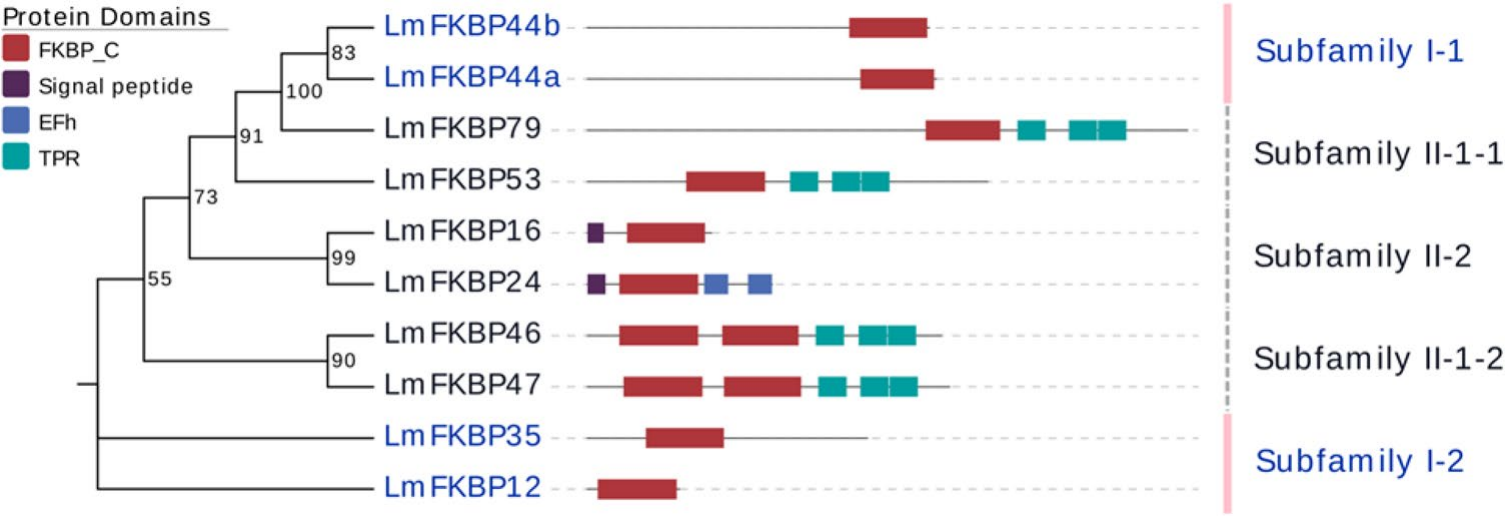
Analysis of phylogenetic tree and conserved domains of FKBPs in *L. migratoria*. Protein domains: FKBP_C (FKBP-type peptidyl-prolyl cis–trans isomerase), EFh (EF-Hand), TPR (Tetratricopeptide repeats). The EvolView was used to map the phylogenetic tree and the structural domain groups association.

Molecular phylogenetic analysis of FKBP family proteins revealed that LmFKBPs form a close association with other organisms (Fig. 1). Meanwhile, the alignment of the amino acid sequences of the FKBP_C domain of the LmFKBPs, was carried out using the FKBP_C domain of *DmFKBP12* as a reference. Among them, both LmFKBP46 and LmFKBP47 had two FKBP_C domains, distinguished as "− 1” and “− 2". The lengths of the domains were between 87–93 amino acids, with the difference among them being less than 7 amino acids. There were 37 positions where the amino acids were relatively similar (Fig. 2). Based on phylogenetic tree and conserved domain analyses, the LmFKBPs were grouped into two subfamilies: subfamily I and subfamily II (Fig. 3). In detail, the sub-family I contained only the FKBP_C domain, and included 4 protein members (LmFKBP12, LmFKBP35, LmFKBP44b and LmFKBP44a); the subfamily II contained FKBP_C domain and other domains (tetratricopeptide repeats (TPR), EF-hand (EFH)), including 6 protein members (LmFKBP16, LmFKBP24, LmFKB46, LmFKBP47, LmFKBP53 and LmFKBP79). In addition, the two subfamilies were further subdivided based on the type of protein domains. In terms of the position of the FKBP_C domain, the subfamily I was subdivided into two subclasses (subfamily I-1 and subfamily I-2). The FKBP_C domain of the subfamily I-1 (LmFKBP44b and LmFKBP44a) was located at the C-terminus, while the subfamily I-2 (LmFKBP12 and LmFKBP35) was situated elsewhere. Based on whether it contained the TPRdomain, the subfamily II was subdivided into two subclasses (subfamily II-1 and subfamily II-2). The subfamily II-1 (LmFKBP46, LmFKBP47, LmFKBP53, and LmFKBP79) contained TPR. Meanwhile, both LmFKBP46 and LmFKBP47 contained two FKBP_C domains, while LmFKBP53 and LmFKBP79 contained only one FKBP_C domain. The phylogenetic tree also showed a separation between the two subcategories. Therefore, subfamily II-1 was divided into two subcategories (subfamily II-1-1 and subfamily II-1-2). Moreover, the subfamily II-2 (LmFKBP16 and LmFKBP24) contained no TPR, but all had the signal peptide structure. Although LmFKBP24 contained an additional EFH domain, the resulting tree showed that it was closely related to LmFKBP16.

### Developmental expression patterns of *LmFKBPs*

**Figure 4:**
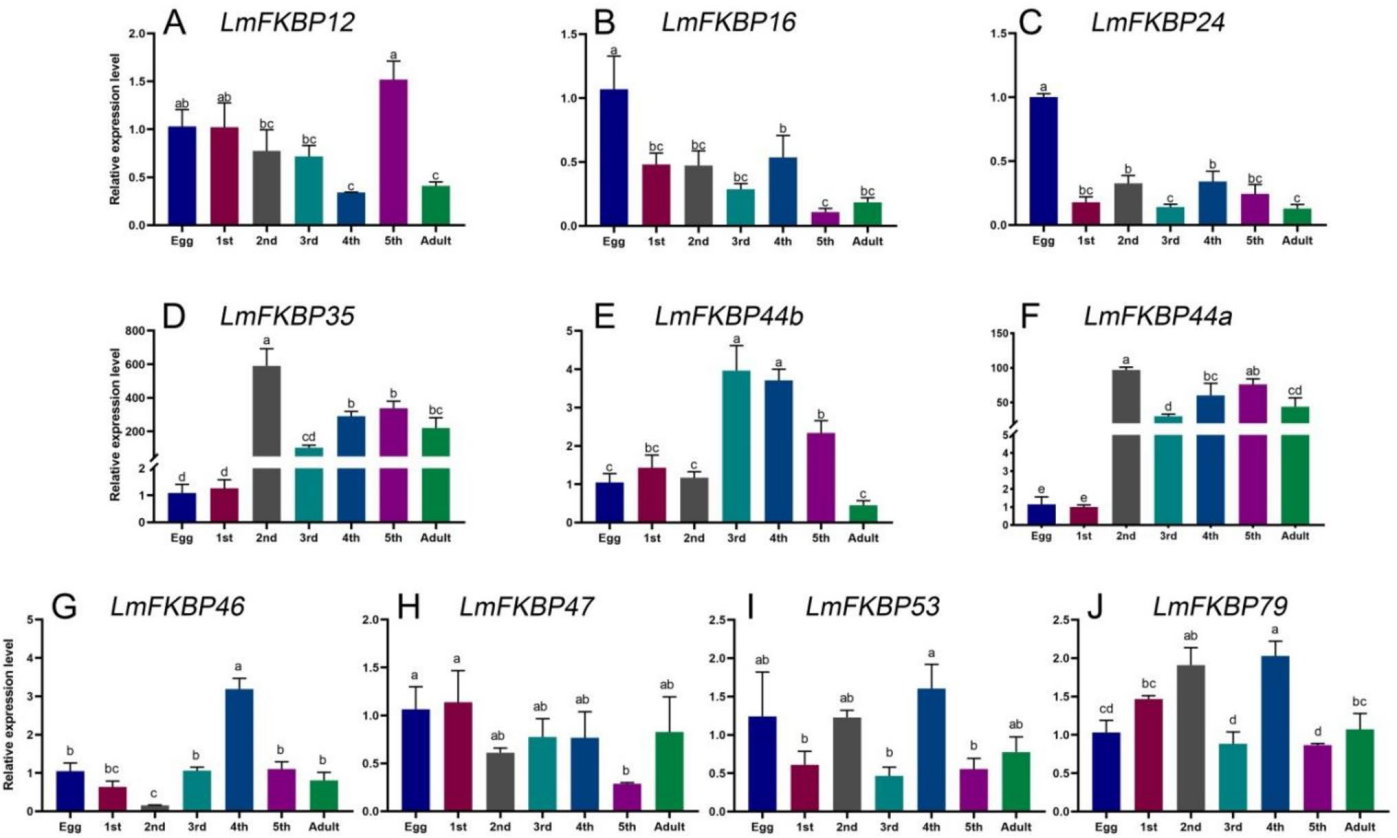
Expression patterns of 10 *FKBPs* at different developmental stages of *L. migratoria*. Bars represent Mean ± SEM (standard error of mean) (n = 3). The genes expressions at the egg stage were taken as the control. Different lowercase letters indicate significant differences in relative expression levels (*P* < 0.05). The X axes in (**A**–**J**) show the analyzed developmental stages of *L. migratoria*, including eggs and nymphal instars (1st + 2nd = first and second instars, 3rd = third instar, 4th = four instar, 5th = fifth instar). (**A**–**J**) are the relative expression levels of *LmFKBP12*, *LmFKBP16*, *LmFKBP24*, *LmFKBP35*, *LmFKBP44b*, *LmFKBP44a*, *LmFKBP46*, *LmFKBP47*, *LmFKBP53*, *LmFKBP79* at different developmental stages.

The expression patterns of the 10 *LmFKBP* genes at different developmental stages (including eggs, 1st, 2nd, 3rd, 4th, and 5th instar stages and adults) were determined by RT-qPCR (Fig. 4). The results showed that 10 *LmFKBPs* were expressed throughout the developmental period, but the expression pattern of each *LmFKBP* was different. For instance, the expression level of *LmFKBP12* gradually decreased from the egg stage to the 4th instar stage, but increased sharply to its highest level at the 5th instar stage, and then decreased again at the adult stage (Fig. 4A). The highest expression levels of *LmFKBP16* and *LmFKBP24* were at the egg stage (Fig. 4B,C). *LmFKBP35* and *LmFKBP44a* had similar expression patterns, with higher expression levels from the 2nd instar to adult stage, but lower expression levels from the egg stage to the 1st instar stage (Fig. 4D,F). Likewise, the expression patterns of *LmFKBP44b* and *LmFKBP46* were also similar; their expression levels were higher in the 3rd to 5th instar stage, but lower in the other stages (Fig. 4E,G). Furthermore, the expression level of *LmFKBP47* was higher in the egg and the 1st instar stage, but lower in the other stages (Fig. 4H). Interestingly, *LmFKBP53* and *LmFKBP79* genes showed higher expression at the 4th instar stage, but lower in the other stages (Fig. 4I,J).

### Expression patterns of *LmFKBP* genes in different tissues

**Figure 5:**
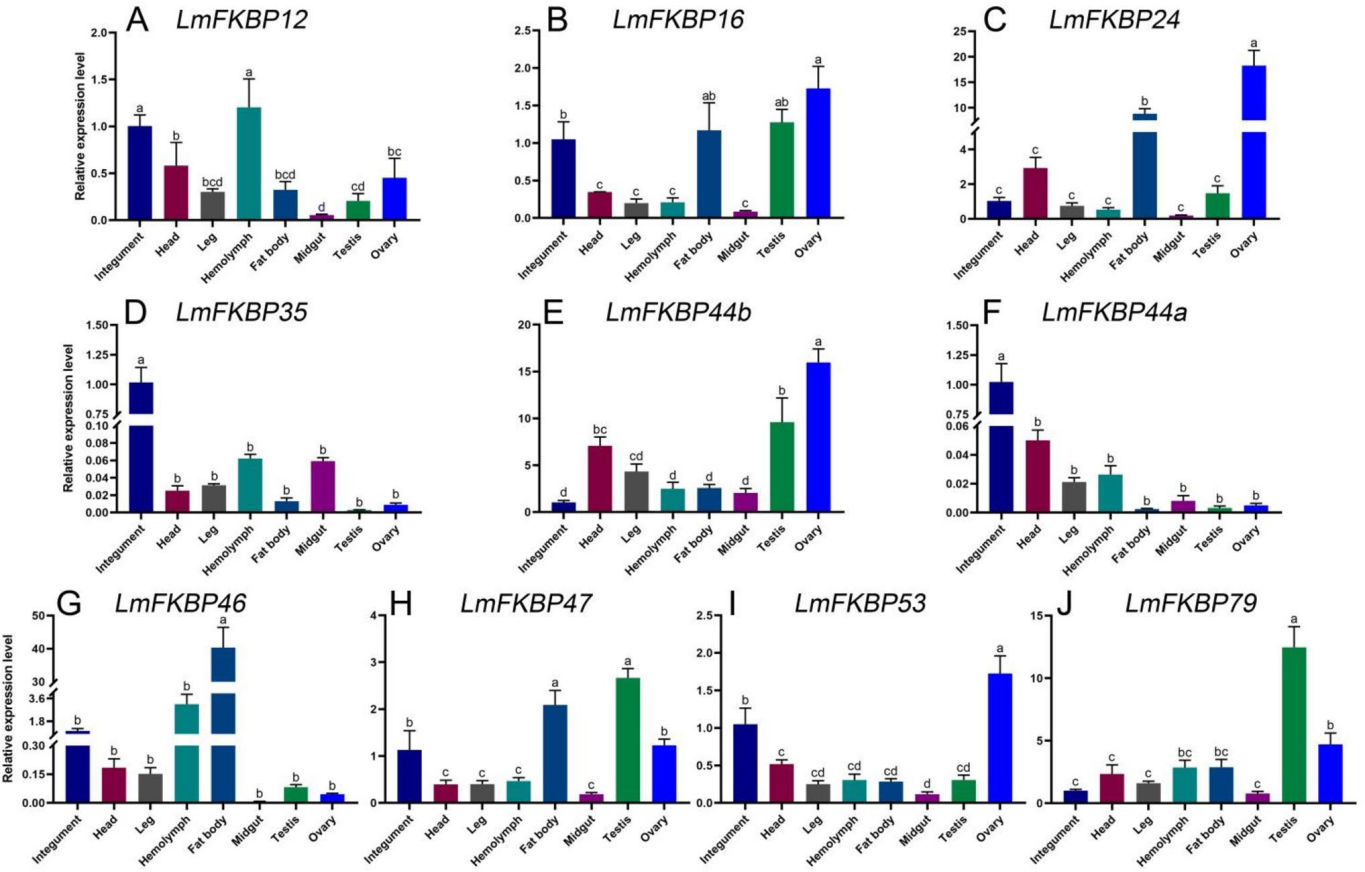
Expression patterns of 10 *FKBPs* in different tissues of *L. migratoria*. Bars represent Mean ± SEM (standard error of mean) (n = 3). The genes expressions in the integument were taken as the control. Different lowercase letters indicate significant differences in the relative expression levels (*P* < 0.05). The X axes in (**A**–**J**) show the analyzed tissues of *L. migratoria*, including integument, head, leg, hemolymph, fat body midgut, testis, ovary. (**A**–**J**) are the relative expression levels of *LmFKBP12*, *LmFKBP16*, *LmFKBP24*, *LmFKBP35*, *LmFKBP44b*, *LmFKBP44a*, *LmFKBP46*, *LmFKBP47*, *LmFKBP53*, *LmFKBP79* in different tissues.

The expression patterns of the 10 *LmFKBPs* in different tissues (including integument, head, leg, hemolymph, fat body, midgut, testis, and ovary) were also determined by RT-qPCR (Fig. 5). The 10 *LmFKBPs* were expressed in all tissues, but their expression patterns differed. Taking the expression of *LmFKBPs* in the integument as the reference, *LmFKBP12* was highly expressed in the hemolymph, integument, head and ovary, but lowly expressed in the leg, fat body, testis and midgut (Fig. 5A). *LmFKBP16*, *LmFKBP24*, *LmFKBP44b*, and *LmFKBP53* all showed their highest expression in the ovary (Fig. 5B,C,E,I). Among them, the expression of *LmFKBP16* was relatively high in the testis, fat body and integument, and *LmFKBP24* was relatively high in the fat body and head. In addition, the expression of *LmFKBP44b* was relatively high in the testis and head, and *LmFKBP53* was relatively high in the integument. *LmFKBP35* and *LmFKBP44a* had similar expression patterns, with their highest transcriptional levels recorded in the integument, but lower in other tissues (Fig. 5D,F). *LmFKBP46* was abundantly expressed in the fat body (Fig. 5G). *LmFKBP47* was mainly expressed in the testis, fat body, integument, and ovary (Fig. 5H), whereas *LmFKBP79* was highly expressed in the testis and ovary (Fig. 5J).

## Discussion

FKBPs are evolutionarily conserved proteins with PPIase activity. They are widely involved in different biochemical processes, such as transcription, protein folding, receptor signaling, and immunosuppression^26^. Various numbers of FKBPs have been identified in the genomes of *Saccharomyces cerevisiae* (4), *Drosophila melanogaster* (8), *Caenorhabditis elegans* (8), *Arabidopsis thaliana* (23), *Oryza sativa* (29), *Mus musculus* (18) and *Homo sapiens* (18)^27–30^. In this study, we applied bioinformatics techniques to identify 10 *FKBPs* from the transcriptome and genome of *L. migratoria*. Based on the prediction of conservative domains and phylogenetic tree analysis, the *LmFKBP* family was classified into two subfamilies and five subclasses.

FKBPs not only contain conserved FKBP_C domain, but also contain other domains such as EFH and TPR^31^, which are involved in many biological processes. For example, the *Homo sapiens HmFKBP12*, which contains only the FKBP_C domain, was reported to participate in protein folding, bind to the immunosuppressive drugs, FK506 and rapamycin, then interact with CaN and mTOR, to regulate signal transduction, and play a key role in immune suppression^32–34^. Among insects, *Bombyx mori BmFKBP12B* was reported to express in the whole developmental stages and in different tissues, then participated in the regulation of silkworm embryonic development^16^. *Aedes aegypti AeFKBP1* was demonstrated to mediate resistance to DENV (Dengue virus)^18^. In our study, *LmFKBP12* was expressed at different developmental stages and tissues of *L. migratoria*, with the highest expression recorded in the hemolymph. Their expression levels in the egg and ovary were also relatively high. These results indicated that *LmFKBP12* may be involved in the regulation of immunosuppression, embryonic development or diapause in *L. migratoria*.

Recent research has shown that FKBPs with the same domain may play diverse roles in various organisms. For instance, *HmFKBP22*, which contained the EFh domain, has been shown to bind calcium ions^35^, while *DmFKBP14* containing the EFh domain was proved to be involved in the development of eyes and wings in *D. melanogaster*^17^. In our study, we also identified the *LmFKBP24* to contain the EFh domain. Spatiotemporal expression analysis showed that it was expressed at all development stages, and was highly expressed in the ovary and fat body. The fat body is the functional equivalent of the mammalian liver and plays a pivot role in insect immune regulation, while the ovary is an important organ involved in insect reproduction development. Therefore, we speculated that the *LmFKBP24* may participate in the regulation of embryonic development and immune process. Moreover, we are on the way of this research.

The TPR domain has been reported to be involved in protein interactions with the ability to cooperate with heat shock proteins, Hsp70/Hsp90. The protein complex pro-motes correcting protein folding and plays an important role as molecular chaperones^36,37^. *LmFKBP46* and *LmFKBP47* were highly expressed in the fat body, while *LmFKBP53* and *LmFKBP79* were mainly expressed in the testis and ovary in *L. migratoria*. Meanwhile, both *LmFKBP46/47* and *LmFKBP53/79* contained the TPR domain, but the two subclasses contained different numbers of FKBP_C domains, indicating that the additional FKBP_C domains may affect their functions in different tissues. However, this needs a further verification.

Some FKBPs contain nuclear nucleoplasmin-like (NPL) domain can interact with both nucleosomes and the small subunit processome^38^. For example, *DmFKBP39*, which contains the NPL domain, is located in the nucleus and has been shown to bind to DNA and participates in the regulation of transcription signals^11^. A recent study showed that *DmFKBP39* expresses at a high level and controls juvenile hormone (JH) activity at the larval stage^39^. Our analysis showed that the *LmFKBP44b* contains a NPL domain (not shown in Fig. 3) and was located in the nucleus. This indicated that its function may be similar to that of the *DmFKBP39*.

## Conclusions

Here, we identified and classified 10 *LmFKBP* genes from *L. migratoria*. As a crucial first step toward understanding their functions, a comprehensive examination of the expression patterns of *LmFKBPs* genes was performed. We made a clear picture on the expression levels of 10 *LmFKBPs* in the fat body, hemolymph, testis, and ovary which would help accelerating the exploration of this gene family. Based on available knowledge, *LmFKBPs* may play important roles in immune regulation and embryonic development of *L. migratoria*, which will be studies in the following researches.

## Supplementary Information

Supplementary Table S1.

## Data Availability

The datasets generated or analysed during the current study are available from the corresponding author on reasonable request.
